# Successful management of a pancreatic mature cystic teratoma with Whipple procedure: A case report

**DOI:** 10.1016/j.ijscr.2024.110504

**Published:** 2024-10-23

**Authors:** Iyad Al Jada, Maaweya Jabareen, Wasef Alhroub, Majd Oweidat

**Affiliations:** a Department of Surgery, Hebron University, Hebron, Palestine; b Faculty of Medicine, Hebron University, Hebron, Palestine

**Keywords:** Mature cystic teratoma, Pancreas, Dermoid cyst, Whipple procedure, Case report

## Abstract

**Background:**

Mature cystic teratomas of the pancreas, also known as dermoid cysts, are exceptionally rare tumors characterized by well-differentiated parenchymal tissues. Typically containing diverse tissues from all three germ layers, these teratomas are most commonly found in the ovaries and testes, with infrequent occurrences in the pancreas.

**Case presentation:**

A 30-year-old male with type 2 diabetes mellitus presented with elevated liver enzymes and serum CEA levels. A CT scan detected an 8.8 × 7.2 cm retroperitoneal mass with calcifications. Due to the tumor's involvement with critical structures, a Whipple procedure was performed. Post-surgery, the tumor was confirmed to be a mature cystic teratoma, and the patient experienced a smooth recovery.

**Discussion:**

Pancreatic teratomas are rare, typically affecting younger patients and predominantly occurring in the body or head of the pancreas. These tumors, often categorized into mature and immature types. Diagnosis relies on imaging techniques such as ultrasound, CT, and MRI, which reveal key features like fat, calcifications, and fat-fluid levels. Differential diagnoses include various pancreatic cystic lesions. Surgical resection is the primary treatment, and this case highlights the diagnostic challenges and the critical role of imaging in guiding surgical decisions.

**Conclusion:**

This report describes a rare case of a pancreatic mature cystic teratoma, one of only 52 documented cases. Despite the absence of significant symptoms, imaging revealed a large mass, and Whipple procedure was performed due to its complex relationship with vital structures. This case illustrates the diagnostic and therapeutic challenges associated with such rare tumors.

## Highlights

• Teratomas are neoplasms originating from tissues derived from all three germ cell layers.
• Pancreatic teratomas are an exceptionally rare occurrence.
• Diagnosing pancreatic teratomas is challenging and relies heavily on imaging modalities and pathological analysis.
• A comprehensive set of differential diagnoses must be considered when evaluating pancreatic teratomas.

## 1 Introduction

This work has been reported in line with the SCARE criteria [1].

A mature cystic teratoma of the pancreas is an uncommon condition characterized by the presence of well-differentiated parenchymal tissues. Typically composed of ectodermal components, these teratomas are frequently referred to as dermoid cysts [2]. They can include a variety of tissues derived from all three germ layers, such as teeth, bone, cartilage, hair, and dermal appendages like sweat glands, sebaceous material, and hair follicles [3].

Macroscopically, these cysts often present with a thick, well-defined wall and a pasty yellow caseous appearance, rather than being clear and serous. Microscopically, they exhibit distinct components from one or more germ layers [4]. While dermoid cysts are commonly found in the ovaries and testes, their occurrence in the pancreas is very rare, with limited cases reported in the literature [5].

In this report, we present a rare case of pancreatic mature cystic teratoma. To the best of our knowledge, this is only the 53rd case reported in the literature, underscoring its rarity. This case not only highlights the diagnostic challenges associated with pancreatic teratomas but also illustrates the successful management of this uncommon condition.

## 2 Case presentation

A 30-year-old male with a history of non-insulin-dependent diabetes mellitus (NIDDM) presented to a public health care center for routine follow-up. During this visit, elevated liver enzymes were detected, with ALT at 166 U/L and ALP at 116 U/L. Notably, the patient reported no abdominal symptoms such as pain, nausea, or changes in bowel habits. Serum carcinoembryonic antigen (CEA) levels were elevated at 5.3 ng/mL, while serum alpha-fetoprotein (AFP) and carbohydrate antigen 19-9 (CA19-9) levels were within normal ranges.

Given these findings, further diagnostic evaluation was warranted. An abdominal ultrasound was performed, revealing a heterogeneous mass in the midline retroperitoneum. The mass measured approximately 8.8 × 7 cm, with a calcified component and indistinct margins relative to the head of the pancreas. The ultrasound suggested the presence of a significant retroperitoneal mass but did not provide a definitive diagnosis.

**Fig. 1:**
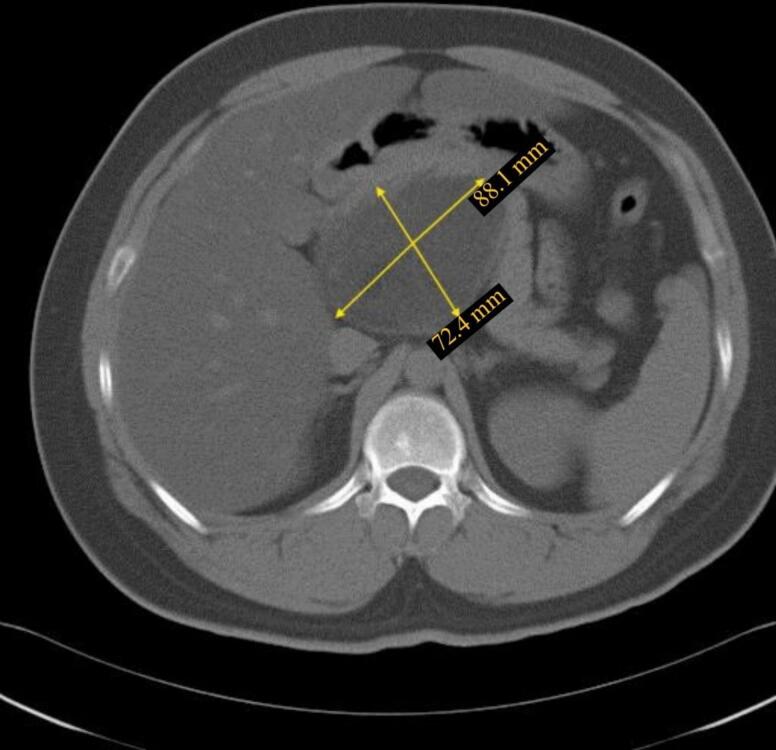
The axial CT scan without IV contrast revealed an 8.8 × 7.4 cm well-defined retroperitoneal solid hypodense mass located posterior to the head of the pancreas.

**Fig. 2:**
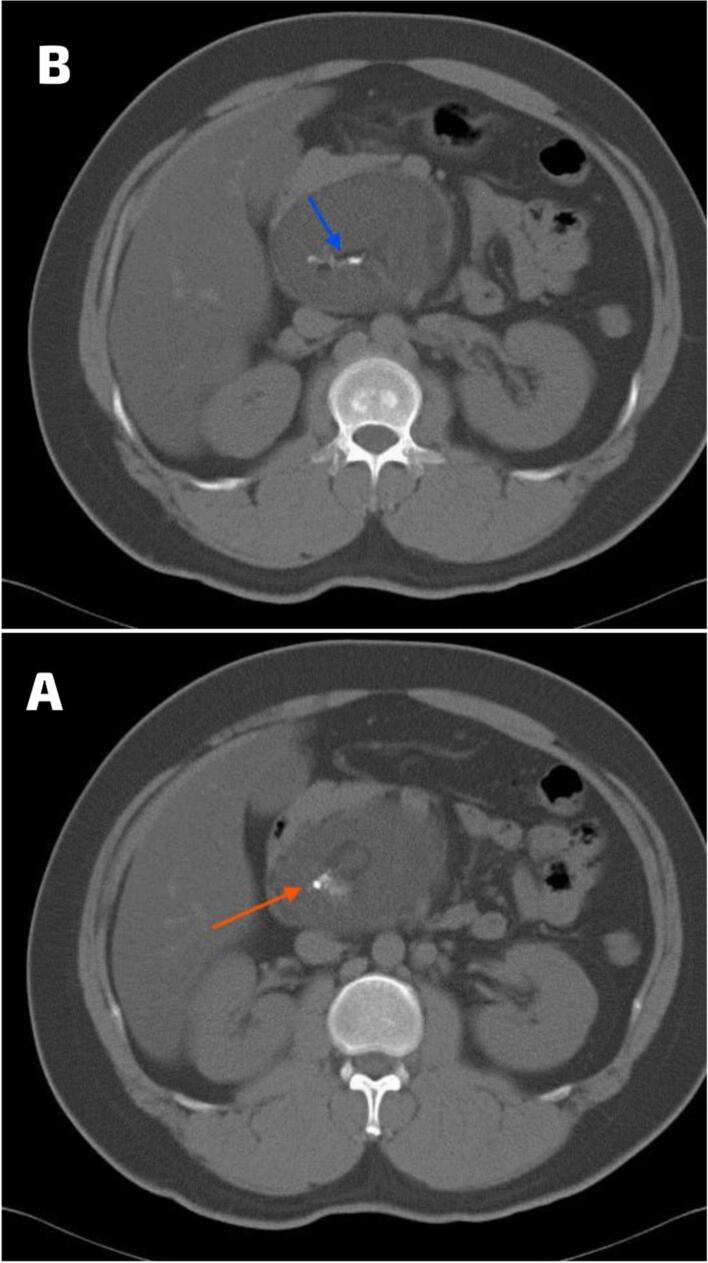
The axial CT scan without IV contrast revealed: A. multiple calcified areas at the center (red arrow); and B. a small area of fat density (blue arrow). (For interpretation of the references to colour in this figure legend, the reader is referred to the web version of this article.)

A subsequent CT scan without IV contrast was conducted, which identified an 8.8 × 7.2 cm well-defined retroperitoneal solid hypodense mass posterior to the head of the pancreas (Fig. 1). The mass showed multiple spots of calcification at the center, in addition to a small area of fat density (Fig. 2). Despite these imaging findings, there was no clear demarcation between the mass and adjacent pancreatic structures.

Given the size and imaging characteristics of the lesion, and in the absence of a preoperative biopsy, a decision was made to proceed with surgical intervention. During the operation, the tumor was found to be located posteriorly and was adherent to critical structures, including the superior mesenteric artery, portal vein, and the head of the pancreas. Additionally, the tumor was noted to be in close proximity to and encasing the common bile duct (CBD). Using both sharp and blunt dissection techniques, we carefully removed fibrous tissue and adherent bands while preserving the integrity of the blood vessels, ensuring no local invasion of the superior mesenteric artery and portal vein. A harmonic scalpel was employed to facilitate precise cutting and coagulation, minimizing tissue trauma and bleeding.

Due to the tumor's significant involvement with major vascular structures and its encasement of the CBD, the surgical team opted for a Whipple procedure. This extensive approach was necessary to achieve complete resection of the tumor and to address the complexities of its anatomical relations.

**Fig. 3:**
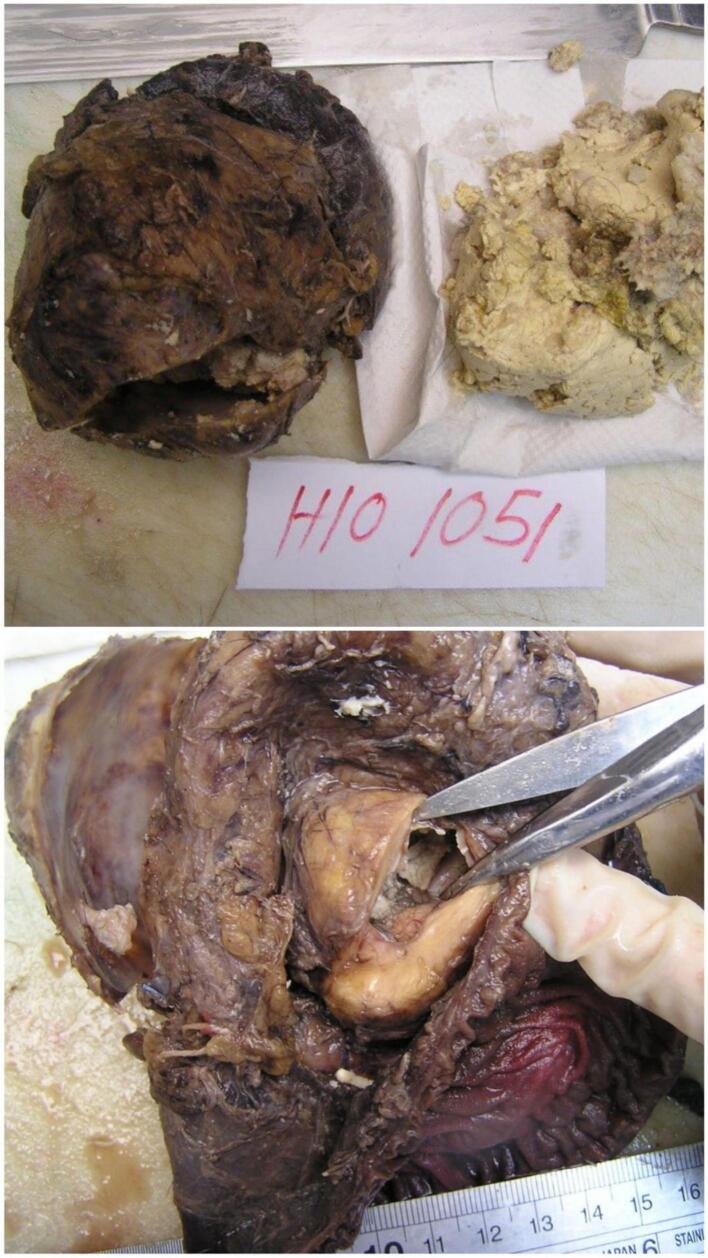
Postoperative image of the resected tumor demonstrating its contents: teeth, bones, hair, and fat, characteristic of a mature cystic teratoma.

Postoperatively, the resected tumor was found to contain teeth, bones, hair, and fat, as shown in (Fig. 3). Histopathological examination confirmed the diagnosis of a mature cystic teratoma. The patient recovered well from the surgery and was closely monitored for any potential postoperative complications.

## 3 Discussion

Cystic teratomas, unusual tumors originating from germ cells, predominantly appear in the ovaries, testes, skull, brain, mediastinum, retroperitoneum, momentum, and bladder. It is exceptionally uncommon for the pancreas to be the primary location for these tumors. The first description of a mature cystic teratoma in the pancreas was made by Kerr in 1918 [6,7].

Teratomas are a type of neoplasm that can be classified into two main categories: mature and immature. Mature teratomas are further subdivided into solid and cystic types, with the cystic variety often referred to as a “dermoid cyst.” While dermoid cysts are predominantly observed in the ovaries, they can also develop along the trajectory of ectodermal cell migration, commonly appearing in the midline regions of the body [8]. Histologically, a dermoid cyst is characterized by a well-defined wall lined with stratified squamous epithelium. The lumen of the cyst often contains mature adnexal structures of mesodermal origin, including hair follicles and shafts, as well as sebaceous and eccrine glands [9].

Pancreatic mature teratomas are documented to occur predominantly in younger patients with an average age of 34.7 years and show a slight male predominance [10]. These tumors are most commonly located in the body of the pancreas, accounting for 35.6 % of cases, followed by the head at 33.3 %. The tail is involved in 15.5 % of cases, while the body/tail combination represents 8.9 %, and the head/body combination accounted for 6.7 % [11]. The average tumor size is approximately 8 cm, with reported sizes ranging from 2.2 cm to 25 cm [11,12]. As of 2020, the literature has reported around 50 cases of pancreatic teratomas. The clinical presentation of a mature pancreatic teratoma is frequently nonspecific and may involve vague gastrointestinal symptoms, such as diffuse abdominal pain, dyspepsia, nausea, back pain, or weight loss [13]. During physical exams, a palpable abdominal mass is frequently noted in cases of enlarged dermoid cysts [14].

The diagnosis of retroperitoneal teratoma depends on clinical presentation, laboratory investigations, and mainly on the imaging studies. Tumor markers such as CA 19-9, CA 125, CA 72-4, and CEA, each with differing sensitivities and specificities, are valuable in distinguishing between benign and malignant tumors [15]. When routine laboratory results and clinical findings are inconclusive, diagnosing a mature teratoma primarily depends on imaging modalities such as abdominal ultrasonography (USG), computed tomography (CT), and magnetic resonance imaging (MRI). The characteristics observed in these images are influenced by the relative amounts of different tissue types within the lesion, including fat, fat-fluid levels, and calcifications [16].

Abdominal ultrasonography (USG) shows mature teratomas as hyperechoic lesions with clear margins and high fat content but cannot differentiate them from other soft tissues due to overlapping appearances. A CT scan typically reveals a round, hypodense lesion with a clear boundary and Hounsfield units between −20 and −140 HU, indicative of intratumoral fat. The presence of intratumoral elements, such as fat, fat-fluid levels, and calcification is highly indicative of a mature cystic pancreatic teratoma, making CT more effective than USG for detecting and characterizing these tumors [14]. MRI excels in soft tissue contrast and can differentiate fat-containing lesions through T1-weighted in-phase (IP) and out-of-phase (OOP) imaging. However, to accurately differentiate mature teratomas from other lesions, techniques such as phase-shift GRE and fat suppression are recommended, as they can identify microscopic fat and provide clearer distinctions in signal intensity [14,17].

When suspicion of a pancreatic dermoid cyst arises, several differential diagnoses should be considered. These include pancreatic pseudocyst, serous cystadenoma, mucinous cystadenoma, solid pseudopapillary tumors (SPT), epidermoid cysts in the intrapancreatic accessory spleen (ECIPAS), and lymphoepithelial cysts (LECs). Accurate differentiation among these conditions is essential for effective diagnosis and treatment [14]. The presence of intralesional fat during the imaging evaluation significantly limits the differential diagnosis to liposarcoma, teratoma, cystic lymphangioma, and extramedullary hematopoiesis [18]. In our case, the teratoma was differentiated by elevated CEA levels, which are typically not increased in other differential diagnoses. Additionally, the presence of calcification further supports teratoma as the most likely diagnosis.

Surgical resection is the primary treatment for pancreatic teratomas, though comprehensive surgical guidelines are lacking. Ideally, achieving complete resection without compromising vital structures is crucial, as incomplete removal often results in unfavorable oncological outcomes. The surgical approach is tailored based on the tumor's location, with complete resection being the gold standard for both diagnosis and treatment [5,19]. For example, a study conducted in 2018 presented the case of a 36-year-old female diagnosed with a mature cystic teratoma. Over a span of 30 years, the patient underwent five internal and external drainage procedures in an attempt to resolve the condition; however, these interventions were unsuccessful. Ultimately, a total resection of the tumor was performed, resulting in a favorable outcome. Six months postoperatively, the patient showed significant improvement [11].

While laparoscopic distal pancreatectomy (LDP) typically requires more time than open distal pancreatectomy, it has been shown to provide significant advantages, including reduced blood loss, quicker recovery, and earlier discharge [5]. Complete surgical removal ensures favorable postoperative outcomes, aligning with a near 100 % five-year survival rate after complete resection [20].

In the case presented, a 30-year-old man with non-insulin-dependent diabetes mellitus had elevated liver enzymes and an elevated serum CEA level. Despite the absence of abdominal symptoms, imaging revealed an 8.8 × 7.2 cm retroperitoneal mass with calcified components. The imaging findings—showing a clear fat plane and nodular calcifications—suggested a teratoma. The decision to proceed with surgical resection without a preoperative biopsy was based on these imaging characteristics. Intraoperative findings of the tumor's adherence to critical vascular structures and encasement of the common bile duct led to the decision to perform a Whipple procedure.

This case underscores the diagnostic challenges associated with pancreatic teratomas and highlights the importance of imaging in guiding surgical planning. The presence of calcifications and a fat plane on CT imaging supported the diagnosis of a teratoma and informed the surgical approach. The complexity of the tumor's relationship to surrounding structures necessitated extensive surgical intervention, illustrating the need for comprehensive preoperative assessment and careful surgical planning. A two-year follow-up revealed significant improvement in the patient's condition, with sustained well-being and no complications.

## 4 Conclusion

This report presents a rare case of a pancreatic mature cystic teratoma, underscoring its rarity as only the 53rd case described in the literature. Despite the patient showing no significant symptoms, imaging revealed a substantial retroperitoneal mass. The decision to proceed with a Whipple procedure was guided by the tumor's intricate relationship with critical structures. This case underscores the importance of thorough preoperative imaging and careful surgical planning in managing such rare and challenging conditions. It also highlights the diagnostic challenges posed by the tumor's nonspecific presentation and similarity to other cystic pancreatic lesions.

## Author contribution

Iyad Al jada contributed to writing, editing and reviewing of the original draft with supervision.

Maaweya Jabareen handled conceptualization, data curation, and software.

Wasef Alhroub contributed to the investigation and visualization of resources validation.

Majd Oweidat provided management of resources and validation.

## Informed consent

Written informed consent was obtained from the patient for the publication of this case report and its accompanying images. A copy of the consent form is available for review by the Editor-in-Chief upon request.

## Ethical approval

Ethical approval was not applicable for this study, as our institution's IRB committee at Hebron University does not mandate approval for reporting individual cases or case series.

## Guarantor

Iyad Al jada is the guarantor for this study, taking full responsibility for the research and its outcomes. Iyad Al jada had access to all the data and made the final decision to publish the study.

## Funding

This research did not receive any specific grants from funding agencies in the public, commercial, or not-for-profit sectors.

## Conflict of interest statement

None.

## Data Availability

All data supporting the study's findings are included in the article and are readily accessible.
